# Film-based cultural narrative intervention to enhance mental health literacy and resilience in indigenous adolescents: a mixed-methods co-design study

**DOI:** 10.3389/fdgth.2026.1776792

**Published:** 2026-04-29

**Authors:** Siti Nur Endah Hendayani, Nisha Nambiar, Rathimalar Ayakannu

**Affiliations:** 1 School of Health and Applied Sciences, Lincoln University College, Petaling Jaya, Malaysia; 2 Faculty of Science and Health Technology, University of Jenderal Achmad Yani, Cimahi, Indonesia

**Keywords:** cultural adaptation, film-based intervention, indigenous adolescents, mental health literacy, mixed-methods, resilience

## Abstract

**Background:**

Indigenous adolescents in low- and middle-income countries experience heightened mental health risks while having limited access to culturally appropriate interventions. Mental health literacy, stigma, and resilience are critical determinants of help-seeking and long-term well-being, yet culturally grounded interventions addressing these factors remain scarce.

**Objective:**

This study aimed to co-design and evaluate a culturally sensitive, film-based intervention to improve mental health literacy, reduce stigma, and enhance resilience among Indigenous adolescents in Indonesia.

**Methods:**

This study employed a mixed-methods sequential exploratory design. A 20-minute short film co-created with Indigenous youth and grounded in local narratives was delivered alongside a facilitated group discussion. Seventy-two Indigenous adolescents (mean age 15.4 years; 52.8% female) were assigned to an intervention group (*n* = 36) or a control group (*n* = 36). Post-intervention outcomes were analyzed using ANCOVA, and qualitative feedback was analyzed thematically.

**Results:**

The intervention group showed significant improvements in mental health literacy, substantial reductions in stigma, and moderate gains in resilience compared with controls (*p* < 0.01). Large effect sizes were observed for literacy and stigma, and a moderate effect was found for resilience. Qualitative findings revealed four themes: cultural resonance and identity affirmation, emotional insight and relatability, empowerment and help-seeking motivation, and collective reflection and peer solidarity.

**Conclusions:**

A co-designed, film-based intervention effectively improved mental health literacy, reduced stigma, and enhanced resilience among Indigenous adolescents. This culturally grounded and scalable approach offers a promising strategy for mental health promotion in marginalized communities, warranting further evaluation with larger samples and longer follow-up.

## 1 Introduction

Adolescents living in low- and middle-income countries (LMICs) are increasingly exposed to mental health challenges, driven by a combination of poverty, limited access to health services, and social instability (1–3). This stage of life, which stretches from puberty into early adulthood, is marked by major emotional, cognitive, and social development (1, 2). However, when these developmental transitions occur in environments affected by displacement, family breakdown, or violence, they can increase the likelihood of psychological distress and emotional instability (3). Global data indicate that nearly half of all mental health disorders emerge before age 14 (4), with conditions such as anxiety, depression, and behavioral difficulties being most common. Anxiety disorders alone account for a substantial number of years lived with disability (YLDs) among young people (5), while self-harm, particularly among adolescent girls, remains a significant cause of death globally (6). In Indonesia, estimates suggest that one in three adolescents experience psychological difficulties, and approximately 5% meet the criteria for clinical diagnoses such as anxiety, depression, or behavioral disorders (7, 8).

For Indigenous adolescents, these challenges are often intensified. In addition to the universal risks faced by their peers, they contend with the effects of cultural disruption, historical trauma, and persistent exclusion from health and education systems (9, 10). Although Indigenous peoples constitute a relatively small proportion of the global population, they are overrepresented among those experiencing poverty, marginalization, and systemic inequity (11, 12). In Indonesia, Indigenous communities, commonly referred to as masyarakat adat—live across both rural and urban contexts. Rural Indigenous groups, such as the Baduy in Banten or the Dayak in Kalimantan, often maintain strong traditional governance systems and cultural practices, yet face barriers in accessing modern health care, education, and economic opportunities. Meanwhile, urban Indigenous adolescents, including those who migrate for schooling or work, navigate the dual pressures of preserving cultural identity while adapting to mainstream social norms, frequently encountering discrimination and cultural invisibility (7, 10).

Evidence indicates that suicide, substance use, and emotional distress are disproportionately reported in Indigenous communities (13, 14). These risks are further compounded by gender- and class-based inequalities, with Indigenous adolescent girls particularly vulnerable to mood disorders, anxiety, and stress-related conditions due to intersecting social expectations and limited access to culturally safe support (15, 16). Together, these intersecting disadvantages underscore the urgent need for culturally grounded and community-driven approaches to promote the mental health and resilience of Indigenous youth in Indonesia.

Despite increasing awareness of adolescent mental health challenges, few programs directly address the cultural and developmental needs of Indigenous adolescents. Many existing interventions continue to rely on Western biomedical models that may not align with Indigenous perspectives—such as collective identity, ancestral connection, and spiritual well-being (9, 16). This mismatch often contributes to low participation and mistrust of formal services (7). One potentially impactful yet underutilized strategy is visual storytelling through film, which can communicate complex emotional experiences and culturally relevant messages in accessible and engaging ways (17, 18). Prior studies with mainstream adolescent populations have demonstrated that film-based interventions can increase mental health knowledge and reduce stigma (7, 8, 14). However, their application in Indigenous contexts remains limited, and few efforts have prioritized genuine collaboration with Indigenous youth in designing such tools (10, 12).

At the same time, there is growing recognition that effective mental health strategies for Indigenous youth must be culturally grounded and community-driven. The long-term effects of colonial histories, systemic exclusion, and cultural erasure have disrupted traditional pathways to healing and created persistent barriers to accessing care (9, 11). Services that overlook Indigenous values of holistic well-being, community connection, and spirituality are often perceived as irrelevant or inaccessible (19, 20). Consequently, scholars and practitioners have emphasized the importance of incorporating Indigenous knowledge systems into intervention design, reframing programs not as corrective measures but as tools for empowerment and cultural resilience (10, 21).

Among the various intervention formats, film stands out for its ability to convey emotion, tell stories, and spark reflection (10, 22). As a visual medium, film allows for the expression of cultural narratives in ways that are immersive and relatable. In mental health contexts, film-based approaches may take two primary forms: (1) participants viewing films created for educational or therapeutic purposes, or (2) participants actively co-creating films to express their own narratives. In this study, the intervention emphasized the viewing of a co-designed short film, followed by guided group discussions. Evidence from high-income countries has shown that such film-based approaches can promote awareness, change perceptions, and encourage help-seeking behavior among adolescents (7, 8). However, there is limited research examining how these methods can be adapted for LMIC contexts, and even less exploring their use in Indigenous communities. Some pilot efforts in LMICs, such as participatory theater, digital storytelling, and mobile video education, have demonstrated promising outcomes in enhancing emotional understanding and peer engagement (21). These examples underscore the importance of tailoring content to local realities and using accessible delivery platforms. Yet in Indonesia, particularly among Indigenous youth, film-based and participatory media approaches remain largely untested.

Evidence from adult mental health programs indicates that video narratives can support therapeutic processes by helping individuals reflect on their emotions and reduce internalized stigma (18). Research by Goodwin et al. (23) also supports the use of film in promoting mental health literacy in younger audiences. Still, few studies have explored how these tools might function when shaped by Indigenous cultural values and co-created with the communities they are intended to serve. A participatory approach in this context refers to the active involvement of Indigenous adolescents, elders, educators, and cultural leaders at every stage of intervention development—from identifying community needs, co-creating storylines, and integrating cultural symbols, to piloting and refining the final product. This process ensures that interventions reflect local wisdom, foster ownership, and create meaningful connections, making them more impactful and sustainable over time.

Mental health literacy (MHL) is commonly defined as “knowledge and beliefs about mental disorders which aid their recognition, management, or prevention” (24). More recent conceptualizations expand this definition to include the ability to recognize specific disorders, knowledge of professional and self-help resources, attitudes that facilitate help-seeking, and understanding of risk factors and protective factors (22). For adolescents, MHL plays a crucial role in early identification of symptoms and timely support-seeking.

The selection of mental health knowledge, stigma, and emotional resilience as primary outcomes was grounded in both theoretical and empirical considerations. Mental health literacy is considered a foundational determinant of help-seeking behavior and early intervention, as it shapes adolescents' ability to recognize symptoms and understand when and how to access support (25). Stigma is a critical barrier that prevents youth from engaging with available services; reducing perceived and internalized stigma is essential for increasing openness to care and fostering supportive community environments (26). Finally, emotional resilience represents a protective factor that enables young people to adapt positively to stress, recover from adversity, and sustain psychological well-being (27). This construct is especially vital for Indigenous adolescents, who navigate systemic exclusion alongside cultural transitions. Together, these three interrelated outcomes align with socioecological and resilience theories, which emphasize that strengthening individual knowledge, reducing social stigma, and building coping resources can collectively enhance both personal and community well-being.

Recent culturally responsive prevention models further highlight the importance of integrating social cognition, collective identity, and culturally embedded narratives into adolescent mental health promotion efforts, particularly within collectivist and Indigenous contexts.

This study aimed to develop and examine the impact of a culturally sensitive, film-based intervention designed to improve mental health knowledge, reduce stigma, and enhance emotional resilience among Indigenous adolescents in Indonesia.

## 2 Methods

### 2.1 Study design

**Figure 1:**
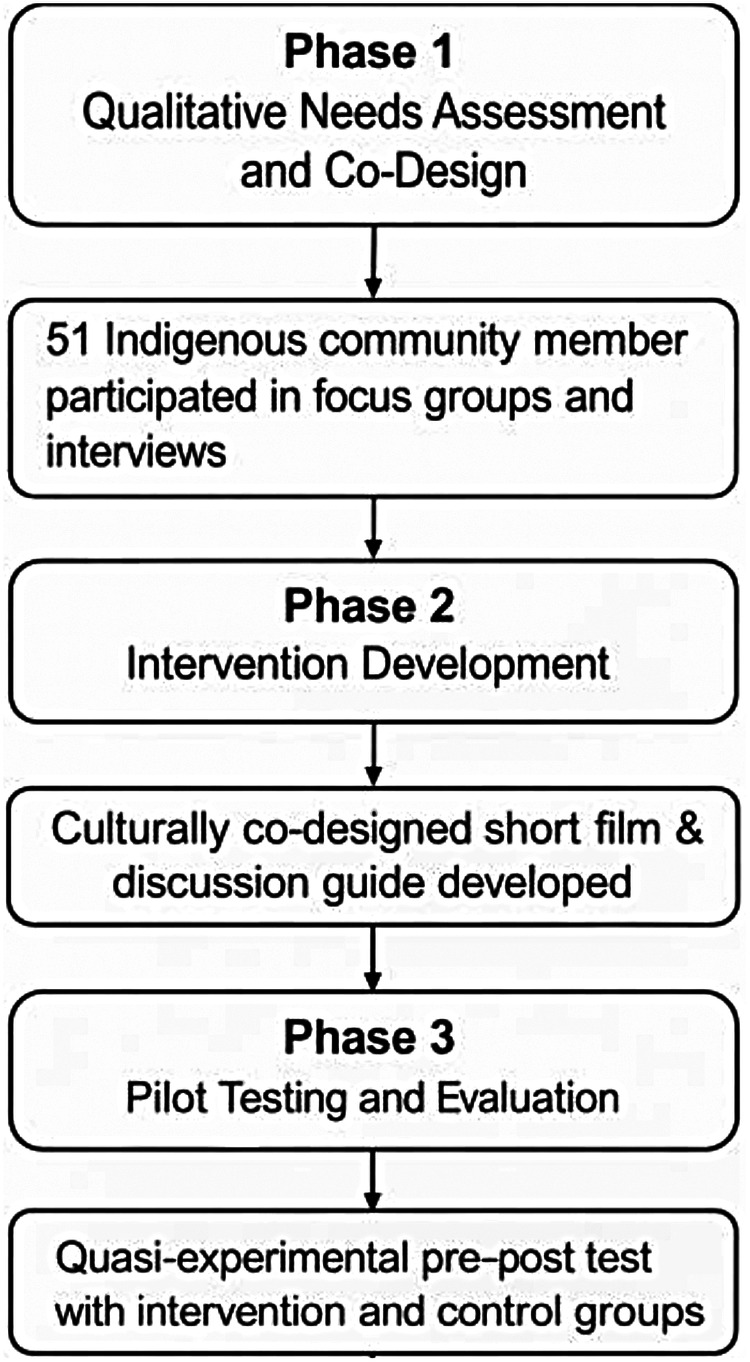
Study flow diagram.

This study adopted a mixed-methods sequential exploratory design that integrated qualitative and quantitative approaches to assess the development, feasibility, and effectiveness of a culturally tailored, film-based mental health literacy intervention for Indigenous adolescents. The study was conducted in three phases: qualitative needs assessment and participatory co-design, intervention development and pilot testing, and quantitative evaluation of impact. The study was not pre-registered due to its exploratory and pilot nature. A visual summary of the phases and participant flow is presented in Figure 1.

### 2.2 Study populations

The study was implemented across four Indigenous communities in West Java, Indonesia, representing both urban and rural contexts. Two rural communities (including the Baduy and Sundanese adat groups) and two urban communities (comprised of Indigenous adolescents who had migrated for schooling or informal work) were purposively selected to reflect the diversity of Indigenous experiences in the region. Selection was guided by prior partnerships with local schools, youth centers, and Indigenous cultural organizations, as well as recommendations from community leaders and local non-governmental organizations working with masyarakat adat. Recruitment took place through a combination of school-based announcements, youth group meetings, and community gatherings organized in collaboration with tribal elders and teachers. Initial contact with each community was facilitated by long-standing collaborations between members of the research team and local health offices, educational institutions, and Indigenous advocacy networks. These established relationships helped to build trust, secure community approval, and ensure that the intervention respected cultural values and protocols. Eligible participants included adolescents aged 13–18 years who self-identified as members of the participating Indigenous groups and were enrolled in either formal schools or alternative learning centers. Proficiency in Bahasa Indonesia and written parental consent were required for participation. Adolescents with cognitive or psychiatric conditions that interfered with comprehension or participation were excluded.

### 2.3 Procedure

#### 2.3.1 Phase 1: participatory exploration and co-design

The first phase focused on engaging key community stakeholders to inform the cultural adaptation and acceptability of the intervention. A total of 51 individuals participated, including 24 adolescents, 10 parents, 8 educators and school counselors, 5 community elders, and 4 healthcare providers.

Participants were recruited purposively with assistance from Indigenous leaders, teachers, and local youth coordinators to ensure diversity across gender, age, and roles within the community. Focus group discussions (FGDs) were conducted separately with adolescents, parents, and educators to encourage open dialogue within peer groups. Individual interviews were held with community elders and healthcare providers, who were considered key knowledge holders and gatekeepers of cultural and health practices.

Data collection was carried out by a team of three trained qualitative researchers with backgrounds in psychology and public health, supported by local facilitators fluent in both Bahasa Indonesia and the respective Indigenous dialects. All researchers completed a two-day training workshop on culturally sensitive interviewing, ethical considerations in Indigenous research, and qualitative data collection techniques. Interviews and FGDs were conducted in neutral and comfortable community settings (e.g., schools, community halls, youth centers) to ensure privacy and accessibility. Sessions lasted between 60 and 90 min. Participation was voluntary, and no monetary compensation was provided; however, refreshments and small stationery packs were offered as tokens of appreciation.

All sessions were audio-recorded with participant consent and transcribed verbatim in Bahasa Indonesia. Where Indigenous terms were used, these were preserved and annotated during transcription to maintain cultural meaning. A bilingual research assistant reviewed transcripts for accuracy. Thematic analysis was conducted following Braun and Clarke's six-step framework. The six phases include: (1) familiarization with the data, (2) generating initial codes, (3) searching for themes, (4) reviewing themes, (5) defining and naming themes, and (6) producing the report (28). A codebook was developed iteratively: two researchers independently coded an initial set of transcripts, compared results, and refined codes through discussion until consensus was achieved. NVivo 14 software was used to manage the data. Intercoder reliability was established by double-coding 20% of the transcripts, achieving a Cohen's kappa of 0.82, indicating substantial agreement. Final themes were refined through team discussions and validated through feedback sessions with a small group of participants and community leaders to ensure cultural resonance.

Insights gathered highlighted local perceptions of mental health, stigma, and resilience, and informed the development of the film narrative. Key elements, such as symbolic imagery, Indigenous language expressions, and culturally specific stressors (e.g., migration for schooling, intergenerational expectations), were incorporated into the storyline to ensure cultural relevance and resonance.

#### 2.3.2 Phase 2: creating the film-based intervention

Building on insights from Phase 1, the second phase focused on the development and pilot implementation of the film-based intervention. Importantly, the intervention did not involve participants creating films themselves; rather, it involved viewing and discussing a co-created short film designed to reflect Indigenous adolescents' experiences.

A 20-minute educational film was co-created through collaboration between Indigenous youth representatives (distinct from the evaluation sample), local artists, mental health experts, and cultural leaders. Youth contributed during storyboarding workshops by suggesting scenarios, dialogue in both Bahasa Indonesia and local dialects, and symbolic imagery that resonated with their lived experiences. Mental health experts integrated evidence-based psychoeducational content, while cultural leaders ensured cultural accuracy and respect for traditions. The storyline centered on themes identified in Phase 1—anxiety management, peer pressure, cultural identity, and coping strategies—expressed through everyday scenarios, traditional attire, and culturally relevant symbols.

Although the film addressed specific topics such as anxiety management and coping, these themes were not measured directly as outcomes because they were intended as pathways to broader psychosocial constructs. For example, by illustrating practical strategies for managing anxiety, the film aimed to increase mental health knowledge; by portraying culturally resonant stories that normalize help-seeking, it sought to reduce stigma; and by highlighting positive role models and coping strategies, it was designed to strengthen emotional resilience. Following the screening, guided discussion sessions (45–60 min) were facilitated by Indigenous youth mentors with support from trained community mental health facilitators. A custom discussion manual structured these sessions around three goals: (1) reinforcing knowledge through open-ended prompts (e.g., “What did you learn about stress from the film?”), (2) addressing stigma through case vignettes that normalized conversations about mental health, and (3) fostering resilience through group activities such as role-play, cultural reflection, and peer support exercises. In this way, the film plus discussion model served as a mechanism for translating narrative themes into measurable outcomes of knowledge, stigma, and resilience.

Feedback during these sessions was captured in three ways, These three feedback mechanisms were refined after Phase 2 and fully implemented during Phase 3 evaluation: (1) detailed field notes by facilitators, (2) anonymized written reflections by participants at the end of the session, and (3) digital feedback submitted through a prototype interface that allowed adolescents to provide anonymous comments. All data were digitized, de-identified, and managed in NVivo 14 for qualitative analysis.

Using thematic analysis, two researchers independently coded feedback from the discussions, identifying recurring themes related to acceptability, cultural resonance, and perceived usefulness. Intercoder reliability was established through iterative comparison and refinement of codes, with discrepancies resolved through team consensus. Themes were validated with Indigenous youth mentors to ensure cultural accuracy.

Findings from Phase 2 informed refinements to the intervention protocol for Phase 3. Specifically, the discussion manual was adjusted to emphasize peer support and cultural pride, additional visual cues were added to enhance accessibility for younger adolescents, and the digital feedback tool was prepared for integration into the evaluation phase.

Facilitators completed a one-day structured training session covering the discussion manual, trauma-sensitive communication, group management skills, and ethical considerations when discussing emotional topics with adolescents.

#### 2.3.3 Conceptual pathway of change

The intervention was theoretically structured around a narrative persuasion framework and resilience theory. The film component primarily targeted cognitive change by enhancing recognition of mental health challenges (literacy). The guided discussion component addressed social and attitudinal dimensions by normalizing help-seeking and challenging stigmatizing beliefs. Together, these processes were expected to strengthen adaptive coping beliefs and perceived social support, thereby contributing to resilience enhancement.

#### 2.3.4 Phase 3: pilot testing and evaluation

The final phase adopted a quasi-experimental pre-post design with intervention and control groups. Schools were purposively selected based on existing partnerships and assigned to intervention or control conditions by location to minimize contamination. Thus, allocation was not randomized at the individual level. This quasi-experimental allocation was selected due to ethical and logistical considerations, including the need to minimize cross-group contamination within small school communities. However, the absence of individual randomization increases the possibility of unmeasured confounding variables, which should be considered when interpreting causal inferences. To avoid contamination across groups, assignment was conducted at the school level rather than the individual level. Four schools were purposively selected based on their location in areas with sizeable Indigenous adolescent populations and existing community partnerships from Phase 1. Two schools were assigned to the intervention arm and two to the control arm.

Schools were approached through community leaders and local education offices, building on relationships established in Phase 1. Each school principal and teachers were briefed about the study's purpose and procedures. Across the four schools, approximately 450 students were enrolled, of whom 92 met the inclusion criteria (ages 13–18, self-identified Indigenous, with parental consent). Seventy-two students were ultimately recruited (36 per group) after exclusions for ineligibility or lack of consent. *A priori* power analysis using G*Power 3.1 indicated a required sample of 64 (f = 0.25, *α* = 0.05, power = 0.80). To allow for attrition, 72 adolescents were enrolled.

Participants assigned to the intervention arm engaged in a two-part activity. First, they viewed a 20-minute co-designed film that was developed in Phase 2 through a participatory process involving Indigenous youth, cultural leaders, educators, and mental health professionals. The film presented culturally relevant narratives and visual symbols addressing common adolescent mental health challenges such as anxiety, stigma, and coping strategies. Following the screening, participants took part in a facilitated group discussion lasting 45–60 min. These sessions were guided by a structured discussion manual created during Phase 2, which included prompts, case vignettes, and small group activities. The discussions were facilitated by trained Indigenous youth mentors and community-based mental health facilitators to ensure cultural safety and relatability. The purpose of the discussion was to help adolescents process the film's messages, share personal reflections, and connect the content to their own experiences (29).

Participants in the control arm did not receive the film-based intervention. Instead, they were provided with standard printed materials on adolescent mental health, including brochures and leaflets that are typically distributed in school health programs in Indonesia. These materials contained general information on mental health awareness, stress management tips, and contact information for local services. This condition was chosen to reflect the usual care available in schools and to provide a baseline for comparing the added value of the culturally tailored, film-based approach.

Intervention fidelity was monitored using a standardized checklist completed by facilitators after each session. The checklist ensured adherence to the discussion manual, duration, key prompts, and group engagement activities. Random spot observations were conducted by a senior researcher to verify protocol consistency. Assessments were administered at baseline and at two weeks post-intervention by trained research assistants fluent in Bahasa Indonesia and the local Indigenous language. Sessions were conducted in classrooms or community centers to ensure familiarity and comfort.

Three culturally adapted instruments were used. Mental health literacy was assessed using an adapted version of the Mental Health Literacy Scale (MHLS) (25). Stigma was measured using an adapted adolescent-focused stigma scale based on Chandra & Minkovitz (26). Resilience was assessed using an adapted version of the Resilience Scale for Adolescents (READ) (27).

All three instruments were culturally adapted for Indigenous adolescents in Indonesia following established cross-cultural adaptation guidelines (30). Minor wording modifications were made to improve age appropriateness and cultural relevance. The pilot sample of 20 adolescents was independent from both the co-design and evaluation samples and was used to test clarity and reliability across all three scales. showed acceptable to high internal consistency, with Cronbach's *α* ranging from 0.78 to 0.87.

Quantitative data were analyzed using SPSS. Descriptive statistics summarized demographics. Between-group differences at post-test were analyzed using analysis of covariance (ANCOVA), controlling for baseline scores. Effect sizes were reported using partial *η*^2^. Within-group changes were examined descriptively.

To complement quantitative findings, qualitative feedback was collected from the intervention group to explore acceptability, cultural resonance, and perceived impact. Immediately after the group discussions, adolescents responded to three open-ended prompts: (1) “What part of the film resonated most with you?”. (2) “What lessons or messages from the film are relevant to your life?”. (3) “What suggestions do you have to improve the program?”. Responses were captured via written reflections and facilitator notes.

Audio recordings of group discussions were transcribed verbatim, and written reflections were digitized for analysis. Two coders independently conducted thematic analysis following Braun and Clarke's framework. A preliminary codebook was developed from Phase 1 themes and refined inductively. Inter-coder agreement was assessed (Cohen's kappa = 0.84, indicating strong reliability). Themes were reviewed with Indigenous youth mentors to ensure cultural validity.

Themes from qualitative feedback, such as cultural resonance, identity affirmation, and peer solidarity provided explanatory depth to the quantitative improvements observed in knowledge, stigma, and resilience. For example, adolescents' reflections on cultural pride and collective coping helped contextualize the significant increases in resilience scores.

### 2.4 Ethical considerations

The study protocol was approved by the Ethics Committee of the Faculty of Science and Health Technology, Universitas Jenderal Achmad Yani (ETIK.098) and conducted in accordance with the Declaration of Helsinki and national regulations. Informed consent was obtained from parents or guardians, with assent from adolescents. Data were de-identified, stored securely, and reported in aggregate with anonymized quotations to ensure confidentiality. No personal identifiers, images, or sensitive materials are included. Participation was voluntary, with no financial or material compensation, and adolescents could withdraw at any time without consequence.

## 3 Results

### 3.1 Participant characteristics

Seventy-two Indigenous adolescents were enrolled in the study, evenly divided between the intervention (*n* = 36) and control (*n* = 36) groups. All participants completed both baseline and follow-up assessments, yielding a 100% response rate. The average age was 15.4 years (SD = 1.5), with females comprising 56.9% of the sample. Most participants (62.5%) lived in rural areas. No statistically significant baseline differences were found between groups in terms of age, gender, residence, or initial scores on mental health literacy, stigma, or resilience (*p* > 0.05), confirming that the groups were comparable prior to the intervention (Table 1).

**Table 1:** Baseline and outcome measures by group (*n* = 72).

Variable	Intervention group (*n* = 36)	Control group (*n* = 36)	*p*-value
Age (mean ± SD)	15.5 ± 1.4	15.3 ± 1.6	0.652
Female (%)	58.3	55.6	0.793
Rural residence (%)	63.9	61.1	0.814
Mental health literacy (pre)	18.1 ± 3.4	17.7 ± 3.8	0.624
Mental health literacy (post)	23.9 ± 3.1	18.2 ± 3.6	<0.001
Mental health stigma (pre)	27.4 ± 5.6	28.1 ± 5.4	0.574
Mental health stigma (post)	21.3 ± 4.9	27.2 ± 5.6	<0.001
Resilience (pre)	56.3 ± 7.8	55.4 ± 7.2	0.693
Resilience (post)	62.1 ± 7.2	56.0 ± 7.3	0.040

*p* < 0.05 is considered significant.

### 3.2 Pilot testing evaluation

Adolescents in the intervention group showed a marked improvement in mental health literacy following the program. Mean scores rose from 18.1 (SD = 3.4) at baseline to 23.9 (SD = 3.1) post-intervention, a statistically significant change (*p* < 0.001). In contrast, the control group's scores remained relatively unchanged (pre: 17.7 ± 3.8; post: 18.2 ± 3.6; *p* = 0.281). Analysis of covariance (ANCOVA) confirmed a significant group difference post-intervention [F(1,69) = 23.47, *p* < 0.001], with a large effect size (*η*^2^ = 0.25). Stigma scores also improved among the intervention group, decreasing from 27.4 (SD = 5.6) to 21.3 (SD = 4.9; *p* < 0.001). The control group, however, did not exhibit meaningful change (pre: 28.1 ± 5.4; post: 27.2 ± 5.6; *p* = 0.204). Between-group analysis showed a significant post-test difference [F(1,69) = 19.61, *p* < 0.001], with a moderate effect size (*η*^2^ = 0.22). Resilience also improved significantly in the intervention group, with scores increasing from 56.3 (SD = 7.8) to 62.1 (SD = 7.2; *p* = 0.002), while the control group remained stable (pre: 55.4 ± 7.2; post: 56.0 ± 7.3; *p* = 0.461). ANCOVA showed a statistically significant difference between groups at follow-up [F(1,69) = 8.77, *p* = 0.004], with a moderate effect (*η*^2^ = 0.11) (Table 2).

**Table 2:** ANCOVA result for post-intervention outcomes.

Outcome variable	Adjusted mean (intervention)	Adjusted mean (control)	F-value	*p*-value	Effect size (*η*^2^)
Mental health literacy (post)	23.7	18.3	23.47	<0.001	0.25
Mental health stigma (post)	21.5	27.1	19.61	<0.001	0.22
Resilience (post)	62.0	56.2	8.77	0.004	0.11

### 3.3 Qualitative feedback

Thematic analysis of open-ended responses from the intervention group (*n* = 36) revealed four major themes (Table 3):

(1) Cultural resonance and identity affirmation

**Table 3:** Theme, sub-theme, and representative quote.

Theme	Sub-theme	Representative quote
Cultural resonance and identity affirmation	Representation of indigenous culture	*The clothes, the way they talk, even the background music—it was all from our daily life. It's rare to see that in school materials.*
	Sense of cultural pride	*Usually, people think our culture is old-fashioned. But here, it felt like something powerful that could help us heal.*
Emotional insight and relatability	Recognition of shared struggles	*When the character cried alone after school, it felt like watching myself. I never thought others could feel the same.*
	Understanding anxiety in context	*I now understand that being anxious isn't just being weak or lazy. It's something real, and it has reasons.*
Empowerment and help-seeking motivation	Shift in attitudes toward seeking help	*Before, I thought I had to solve everything myself. Now I know it's okay to talk to my teacher or even a friend.*
	Increased confidence to support others	*If my friend becomes quiet or angry, I won't ignore it. I'll ask if they're okay like the girl in the film did.*
Collective reflection and peer solidarity	Strengthened peer connection	*When I spoke up during the discussion, others nodded. That was the first time I didn't feel weird for feeling down.*
	Safe space for emotional exploration	*The movie opened the door, but the sharing after helped us walk through it together.*

Beyond simple representation, participants described the film as validating their lived experiences within a broader social context. As reflected in comments about familiar clothing, language, and music, the portrayal of Indigenous cultural elements enhanced authenticity and relatability. Expressions of renewed cultural pride suggest that identity affirmation functioned as an empowering resource, reinforcing collective strength and potentially contributing to resilience gains.

(2) Emotional insight and relatability

Beyond emotional engagement, participants' reflections indicate that the film facilitated emotional recognition and cognitive reframing of distress. Quotes describing identification with characters' struggles and a new understanding of anxiety demonstrate emerging affective awareness, a core component of mental health literacy. By contextualizing emotional difficulties within shared experiences, the narrative appeared to normalize distress and reduce self-blame, thereby mitigating internalized stigma.

(3) Empowerment and help-seeking motivation

Participants' responses reflected a shift from self-reliance toward relational coping. Statements about feeling permitted to approach teachers or peers suggest a transformation in help-seeking attitudes. Additionally, increased willingness to support others indicates that the intervention may have expanded adolescents' perceived social responsibility, reinforcing mutual care as both an individual and collective coping strategy.

(4) Collective reflection and peer solidarity.

Responses under this theme suggest that the guided discussion component strengthened peer connectedness beyond the film itself. Experiences of speaking openly and receiving validating responses highlight the emergence of psychological safety within the group. The discussion space appeared to facilitate shared meaning-making, reducing perceived isolation and fostering solidarity as a protective resilience factor.

Taken together, these qualitative findings provide contextual insight into the quantitative improvements observed in mental health literacy, stigma reduction, and resilience scores. The themes suggest that culturally grounded narrative exposure enhanced cognitive understanding of mental health, while guided peer discussion facilitated emotional processing and social validation. The normalization of distress and increased openness toward help-seeking reflected in participants' narratives align with the measured shifts in attitudes and adaptive coping. Thus, the qualitative data not only corroborate the statistical outcomes but also illuminate the underlying mechanisms—cultural affirmation, emotional recognition, relational agency, and collective solidarity—through which the intervention may have produced its effects.

## 4 Discussion

This study set out to evaluate a culturally co-designed, film-based intervention aimed at improving mental health literacy, reducing stigma, and enhancing resilience among Indigenous adolescents in Indonesia. The findings indicate that the intervention was both effective and well-received. Quantitatively, adolescents in the intervention group demonstrated significant improvements in all primary outcomes compared with controls: literacy scores rose markedly (*η*^2^ = 0.25), stigma levels declined (*η*^2^ = 0.22), and resilience improved (*η*^2^ = 0.11). These results confirm the study hypothesis that a culturally grounded visual storytelling approach could strengthen mental health knowledge while simultaneously reducing negative attitudes and fostering psychological resources. Complementing these outcomes, thematic analysis of participant feedback highlighted four interrelated domains, cultural resonance, emotional insight, empowerment to seek help, and peer solidarity illustrating how the intervention extended beyond knowledge transfer to also promote identity affirmation and social connection. Together, these findings suggest that film-based interventions co-created with communities can offer a scalable, contextually relevant strategy for advancing adolescent mental health in marginalized populations.

This study extends narrative persuasion theory (31) and resilience frameworks (27) by demonstrating how culturally grounded film-based storytelling can shift not only cognitive outcomes (mental health knowledge, stigma) but also socio-emotional capacities (resilience) when co-developed with Indigenous youth. These findings are also consistent with emerging culturally responsive mental health promotion frameworks that emphasize collective identity, social cognition, and contextualized resilience as central mechanisms of change in collectivist societies (32, 33). Such models argue that culturally embedded narratives may strengthen both knowledge acquisition and socio-emotional adaptation by aligning interventions with shared community values. It contributes to bridging Western evidence-based practices with Indigenous knowledge systems, offering a theoretical model of empowerment through culturally resonant media. These findings build on earlier research affirming the positive impact of multimedia-based approaches on mental health awareness (7, 8). What sets this study apart, however, is the integration of community co-design and local cultural elements, such as traditional music, indigenous language dialogue, and symbolic imagery which contributed to a deeper emotional connection with the material and broadened its impact beyond conventional health education formats (9).

Insights from the qualitative feedback further illuminate the intervention's effectiveness. Participants repeatedly described the film as emotionally impactful and reflective of their lived experiences. Many reported feeling seen and understood, reflecting responses consistent with the theory of narrative transportation, which suggests that stories capable of eliciting emotional immersion can enhance personal relevance and understanding (31). The film also appeared to instill a sense of cultural pride, as youth expressed appreciation for seeing their traditions and values represented in a positive and affirming way; something rarely encountered in typical school curricula.

Thematic analysis identified four core outcomes: affirmation of cultural identity, deeper emotional awareness, increased readiness to seek help, and stronger peer connection. These themes highlight the dual function of film as both an educational resource and a tool for psychosocial empowerment. The guided group discussions that followed the screenings reinforced these effects, creating a safe and culturally respectful environment for sharing. This mirrors observations by Chu et al. (34), who emphasized the importance of culturally attuned facilitation in fostering open dialogue within collectivist cultures.

A major strength of this project lies in its co-creation process. By actively involving Indigenous adolescents, local artists, community elders, and mental health practitioners, the intervention was both contextually appropriate and community-driven. This collaborative approach reflects current best practices in cultural adaptation, which stress the value of community involvement and linguistic relevance in improving program impact and sustainability (35, 36).

## 5 Study limitations

Several limitations should be acknowledged. First, the non-random allocation of participants based on geography could have introduced selection bias. Second, the two-week follow-up period limits conclusions about sustained or long-term effects. Third, self-reported data may have been influenced by social desirability bias, particularly in group settings. Fourth, participants' strong engagement may partly reflect the novelty of watching a film in a school health program context rather than the intervention content alone, which raises the possibility that effects could diminish once the activity becomes more routine. Lastly, facilitators and participants were not blinded to group allocation, which may have introduced performance bias. Future studies should therefore incorporate randomized allocation, longer follow-up, and mixed outcome measures (including behavioral or physiological indicators). In addition, testing the intervention across multiple cycles or delivery formats (e.g., mobile platforms) would help determine whether its impact extends beyond the novelty of the format. The short two-week follow-up period prevents conclusions about maintenance of gains, particularly for resilience, which typically develops over longer developmental trajectories.

## 6 Implications for clinical practice and public health

The findings of this study highlight how culturally co-created film interventions can be meaningfully incorporated into educational guidance sessions, youth engagement programs, and broader community health initiatives. Once developed, the cost of delivering these interventions remains relatively low, making them particularly suitable for under-resourced environments. At the policy level, this research emphasizes the importance of promoting mental health strategies that are aligned with the cultural values and everyday experiences of marginalized groups. Programs that are shaped by local languages, traditions, and social realities are more likely to build trust and encourage participation. Moreover, with digital technologies becoming increasingly widespread, there is growing potential to integrate these culturally responsive tools into broader digital health strategies, ensuring that diverse communities have equitable access to inclusive and culturally respectful mental health resources.

## 7 Conclusions

This study offers valuable insights into adolescent mental health promotion by showing that a culturally responsive film-based intervention can effectively improve mental health understanding, reduce stigma, and strengthen emotional resilience in Indigenous youth populations. By using a participatory framework rooted in storytelling, the program was able to connect with learners on both emotional and intellectual levels, thereby enhancing the relevance and accessibility of mental health education. The strong positive feedback from participants highlights the critical role of cultural alignment and active youth participation in intervention design. As mental health concerns continue to escalate among Indigenous adolescents worldwide, delivering culturally grounded content through scalable media platforms represents a meaningful and adaptable strategy for future public health efforts.

## Data Availability

The raw data supporting the conclusions of this article will be made available by the authors, without undue reservation.
